# The Role of Rapid Diagnostic Tests in Managing Malaria

**DOI:** 10.1371/journal.pmed.1000063

**Published:** 2009-04-28

**Authors:** Zeno Bisoffi, Federico Gobbi, Andrea Angheben, Jef Van den Ende

**Affiliations:** 1 Centre for Tropical Diseases, Sacro Cuore Hospital of Negrar, Verona, Italy; 2 Department of Clinical Sciences, Prince Leopold Institute of Tropical Medicine, Antwerp, Belgium

## Abstract

Zeno Bisoffi and colleagues discuss a new clinical trial in Zanzibar comparing symptom-based clinical diagnosis of malaria versus clinical diagnosis plus rapid diagnostic tests.

**Linked Research Article**

This Perspective discusses the following new study published in *PLoS Medicine*:

Msellem MI, Mårtensson A, Rotllant G, Bhattarai A, Strömberg J, et al. (2009) Influence of rapid malaria diagnostic tests on treatment and health outcome in fever patients, Zanzibar—A crossover validation study. PLoS Med 6(5): e1000070. doi:10.1371/journal.pmed.1000070

Anders Bjorkman and colleagues report results from a crossover trial evaluating rapid diagnostic testing for malaria diagnosis in Zanzibar.

In recent years malaria treatment policies have shifted in most African countries to artemisinin-based combination therapies (ACT), which are highly effective but also much more expensive than previous regimens [1]. In order to avoid over-prescription of ACT, new guidelines from the World Health Organization recommend that a laboratory test should be performed before treating [2]. The widespread introduction of rapid diagnostic tests (RDTs) for malaria allows diagnosis to be made even in health settings lacking any laboratory facility. Since RDTs generally cost less than a full course of ACT, their introduction should not only improve malaria management but should also limit malaria treatment costs.

However, recent research has shown that clinicians are reluctant to refrain from treating for malaria after a negative test [3],[4]. Clinicians' adherence to a test-based strategy is a key factor in determining whether the strategy is effective at improving management and curtailing costs [5],[6]. If the result of a test is not going to influence management, then doing the test is a waste of money [6]. Moreover, RDT-based policy is usually restricted to adults and older children, while for under-fives presumptive malaria treatment of all fevers is still recommended [2]. This leads to the paradox that the improved diagnostic techniques are of no utility for the group at highest mortality risk.

## Test-Based Malaria Management in Children

*PLoS Medicine* recently published a provocative debate on whether or not presumptive treatment in under-fives should be abandoned. Valérie D'Acremont and colleagues argued that it is high time to move to laboratory-confirmed (usually RDT-based) diagnosis for younger children [7]. They noted that malaria incidence is declining in many countries, and so is the probability that an African child with fever has malaria—thus treating all fevers in children as malaria would lead clinicians to miss other, potentially fatal, causes. Mike English and colleagues replied that the reported sub-optimal sensitivity of RDTs in routine use means that use of RDTs risks missing true malaria cases [8]. Moreover, they argued, the poor adherence of health professionals to a test-based management strategy might not be easy to solve just by training.

The divided opinion on this topic reflects a lack of evidence. There is not yet enough evidence for the safety of a test-based strategy for young children, and there is no evidence that satisfactory adherence to a test-based management strategy can be achieved by training. Operational research is needed to fill in these evidence gaps [6]. A new study in this issue of *PLoS Medicine*, by Anders Bjorkman and colleagues, addresses these research priorities [9].

## A New Study on Malaria Management Based on RDTs

Bjorkman and colleagues conducted a cross-over clinical trial of symptom-based clinical diagnosis (CD) versus CD plus RDT in four primary health care facilities in Zanzibar. Patients of all ages with reported fever lasting 48 hours were eligible, and they were allocated on alternate weeks to CD alone or CD+RDT. Follow-up was 14 days, and ACT was prescribed to patients diagnosed with malaria in both groups. Malaria management was provided by registered nurses, all of whom had been trained in malaria case management. The RDT was a histidine-rich protein 2–based test called Paracheck.

The use of RDT had a major impact on clinical decisions. In children, the prescription rate of antimalarial treatments in the CD+RDT group was half that observed in the CD group (42% versus 84% and 44% versus 86% for children under five years and five to 15 years, respectively). In adults, the difference was even more striking (16% versus 87%). Although the authors do not give details about specific decisions taken according to RDT results, this difference suggests that the compliance was very high, as most RDT-negative patients were not treated for malaria. The prescription rate of antibiotics was higher in the CD+RDT group than in the CD group, suggesting that negative malaria RDT results led the nurses to consider and treat alternative causes of fever.

No death was recorded in either group at 14 days of follow-up. The authors do not specify if they observed a difference in frequency of severe complications and/or of referral to a higher level of care; however, the patients in the CD+RDT group were less likely to re-attend the clinic during the follow-up period.

## Implications for Malaria Management Policies

Are these results generalisable—do they suggest that a test-based policy should be recommended even for young children? We will briefly examine the main issues: adherence, safety, and cost.

For the purpose of this study, nurses were trained for one day only. In two of the four health centres, RDTs were introduced for the first time. The excellent adherence to test-based management seen in the trial is surprising, and contrasts with previous studies [3],[4],[10], as well as with our recent findings in Burkina Faso [11]. In our study, even after a three-day training, adherence remained very poor: 80% of RDT-negative febrile patients were treated for malaria in the dry season, and 85% in the rainy season [11]. There are many possible reasons to explain the striking difference between Bjorkman and colleagues' results and those seen in other studies (including our study). We believe that a major reason for the difference was that in Bjorkman and colleagues' study, the research assistants were *also* the prescribers, having to record their own prescribing behaviour, which may have influenced their decisions. The very encouraging adherence rates in this new study should be corroborated by further research on teaching and supervision strategies aimed at achieving satisfactory compliance in real-life situations.

As far as safety is concerned, Bjorkman and colleagues' study indicates that the RDT-based management strategy is safe for both adults and children. The study found no severe malaria case after a missed malaria treatment following a false negative result. Moreover, the lower rate of re-attendance in the CD+RDT group suggests a better clinical outcome. Nevertheless, definitive evidence on safety can only be found by a study that is adequately powered to detect differences in severe clinical outcomes, including deaths, and/or by pooling results obtained in different study settings [11].

Are RDTs cost-effective? Bjorkman and colleagues found that overall cost was the same with CD as with CD+RDT. However, the test-based strategy was associated with cost savings in adults (overall cost US$2.74 with CD+RDT versus US$3.02 with CD alone), due to the much lower proportion of malaria treatment in this group. Since Bjorkman and colleagues conducted their trial, malaria incidence in Zanzibar has further declined [12], and the test-based strategy would probably become even more cost-effective should this decline persist.

## Conclusion

This new study brings additional arguments, albeit no definite evidence, in favour of extending an RDT-based malaria management strategy to young children, in a situation of moderate- to low-intensity malaria transmission. This strategy would, however, be justified only if adherence to test results were optimal, which remains to be proved in real-life situations. More research is also needed in settings where malaria transmission intensity is higher and/or seasonal variation is greater.

## Abbreviations

ACT: artemisinin-based combination therapy
CD: clinical diagnosis
RDT: rapid diagnostic test

## References

[1] Ogbonna A, Uneke CJ (2008). Artemisinin-based combination therapy for uncomplicated malaria in sub-Saharan Africa: The efficacy, safety, resistance and policy implementation since Abuja 2000.. Trans R Soc Trop Med Hyg.

[2] World Health Organization (2006). WHO guidelines for treatment of malaria.. http://www.who.int/malaria/docs/TreatmentGuidelines2006.pdf.

[3] Reyburn H, Mbakilwa H, Mwangi R, Mwerinde O, Olomi R (2007). Rapid diagnostic tests compared with malaria microscopy for guiding outpatient treatment of febrile illness in Tanzania: Randomised trial.. BMJ.

[4] Hamer DH, Ndhlovu M, Zurovac D, Fox M, Yeboah-Antwi K (2007). Improved diagnostic testing and malaria treatment practices in Zambia.. JAMA.

[5] Lubell Y, Reyburn H, Mbakilwa H, Mwangi R, Chonya S (2008). The impact of response to the results of diagnostic tests for malaria: Cost-benefit analysis.. BMJ.

[6] Bisoffi Z, Van den Ende J (2008). Costs of treating malaria according to test results.. BMJ.

[7] D'Acremont V, Lengeler C, Mshinda H, Mtasiwa D, Tanner M (2009). Time to move from presumptive malaria treatment to laboratory-confirmed diagnosis and treatment in African children with fever.. PLoS Med.

[8] English M, Reyburn H, Goodman C, Snow RW (2009). Abandoning presumptive antimalarial treatment for febrile children aged less than five years—A case of running before we can walk?. PLoS Med.

[9] Msellem MI, Mårtensson A, Rotllant G, Bhattarai A, Strömberg J (2009). Influence of rapid malaria diagnostic tests on treatment and health outcome in fever patients, Zanzibar—A crossover validation study.. PLoS Med.

[10] Zurovac D, Njogu J, Akhwale W, Hamer DH, Larson BA (2008). Effects of revised diagnostic recommendations on malaria treatment practices across age groups in Kenya.. Trop Med Int Health.

[11] Bisoffi Z, Sirima BS, Angheben A, Lodesani C, Gobbi F (2009). Rapid malaria diagnostic tests versus clinical management of malaria in rural Burkina Faso: Safety and effect on clinical decisions. A randomized trial.. Trop Med Int Health.

[12] Bhattarai A, Ali AS, Kachur SP, Mårtensson A, Abbas AK (2007). Impact of artemisinin-based combination therapy and insecticide-treated nets on malaria burden in Zanzibar.. PLoS Med.

